# An Account of Diseases in an Eastern District of London, from June 20, to July 20, 1808

**Published:** 1808-08

**Authors:** 


### *An Account of Diseases in an Eastern District of London, from June 20, to July 20, 1808.*

| | | | |
|---|---|---|---|
| ACUTE DISEASES. | | Hypochondriasis - | 5 |
| Cynanche Tonsillaris | 1 | Hepatitis Chronica - | 3 |
| Ephemera - - | 4 | Fluor Albus - - | 6 |
| Ophthalmia - - | 2 | Menorrhagia - - | 8 |
| Hæmoptysis - - | 4 | Dysuria - - - | 5 |
| Cholera - - - | 3 | Rheumatismus Chronicus | 7 |
| CHRONIC DISEASES. | | PUERPERAL DISEASES. | |
| Tussis - - - | 4 | Ephemera - - | 5 |
| Tussis cum Dyspnœa | 5 | Mastodynia - - | 4 |
| Phthisis Pulmonalis | 3 | Hæmorrhois - - | 3 |
| Hydrothorax - - | 2 | INFANTILE DISEASES. | |
| Anasarca - - | 4 | Ophthalmia - - | 3 |
| Cephalalgia - - | 9 | Tabes Mesenterica - | 1 |
| Vertigo - - - | 4 | Aphthæ - - - | 2 |
| Hemiplegia - - | 1 | Herpes - - - | 4 |

But a few weeks ago, the complaint of a long protracted winter was very general. The wind was blowing from east and north-east almost without interruption, and the cold was sometimes so severe as to expose invalids, and others of delicate health, to very considerable inconveniences. In some parts of June, rheumatic affections were as troublesome, and as liable to recur as in some of the earlier months. Since the beginning of the last week, the temperature of the atmosphere has undergone a most important change. The thermometer has arisen to a height, which is not only uncommon in this climate, but which can hardly be recollected by any person living. A few instances of Cholera have occurred. This disease does not in general appear at so early a period. At the latter end of August, or beginning of September, it more commonly occurs, and we must attribute its more early appearance to the uncommon heat of the weather. The cases, however, referred to in the list, were of the milder kind, and yielded pretty readily to the means which are usually employed.

INTELLIGENCE.

